# “Suiting Up” to Enhance Empathy Toward Aging: A Randomized Controlled Study

**DOI:** 10.3389/fpubh.2020.00376

**Published:** 2020-08-25

**Authors:** Shaun Wen Huey Lee, Pei-Lee Teh

**Affiliations:** ^1^School of Pharmacy, Monash University Malaysia, Bandar Sunway, Malaysia; ^2^Gerontechnology Laboratory, Global Asia in the 21st Century (GA21) Platform, Monash University Malaysia, Bandar Sunway, Malaysia; ^3^School of Pharmacy, Taylor's University Lakeside Campus, Subang Jaya, Malaysia; ^4^School of Business, Monash University Malaysia, Bandar Sunway, Malaysia

**Keywords:** aging suit, simulation, pharmacy, empathy, polypharmacy, aging

## Abstract

**Background:** Healthcare professionals who have a positive attitude and who are more empathetic toward older adults are in a better position to deliver quality healthcare. This study examines the impact of using an aging simulation suit on undergraduate pharmacy students' empathy levels.

**Methods:** One hundred and twenty first-year students enrolled in the Bachelor of Pharmacy course were randomized to either a medication review polypharmacy workshop (control) or an immersive aging simulation suit and medication review polypharmacy workshop (intervention). Intervention participants donned the aging suit and performed a series of tasks, including walking up a flight of stairs and filling up a form to simulate the physical limitations experienced by an older adult. The workshop was delivered at week 10 of semester. Both groups also completed a medication review polypharmacy workshop at week 12 of semester. The primary outcome was a measurement of change on the Jefferson Empathy Scale-Healthcare Professional Questionnaire among both groups at week 12 of semester. Secondary outcomes include the longitudinal impact of intervention after 3 months of the workshop and perceptions on learning.

**Results:** The use of a simulation suit did not increase participants' self-rated empathy compared to control. However, the suit enhanced the ability of participants to understand the physical limitations and visual issues associated with aging. Participants also felt that it enhanced their health advocacy, as it taught them the importance of listening, patience and respect for older adults.

**Conclusion:** The use of an immersive aging suit can be a useful adjunctive tool to help enhance students' understanding of the physical limitations and visual limitations of aging. Further research is needed to understand how these limitations affect other healthcare students.

**Trial Registration:** ClinicalTrials.gov identifier: NCT04133727.

## Introduction

The world's population is rapidly aging—nearly one in every five individuals will be 60 years old and above by 2050, translating to ~2.1 billion people worldwide (1). As a result, many countries worldwide are finding the consequent demand on healthcare systems to be a challenge. In the context of this growing need for healthcare, the World Health Organization has recommended that the care of older adults should be centered around their own concerns and priorities (2). Medical and health science-related schools have now made it their educational mission to make compassionate person-centered care a core value of their curriculum (3–6). However, the skills aspects of training have traditionally received less attention, and most studies (7, 8) have shown that there is a decline in the empathy levels of students that persists beyond training.

Empathy is a predominantly cognitive attribute which involves learning and understanding the experiences, perspective, and concerns of a patient, combined with the ability to resonate with the patient and communicate this perspective to them (9). Such an attribute is an essential skill that is of paramount importance to all individuals, especially to healthcare professionals. Studies have shown that empathetic interactions often lead to increased patient satisfaction, better patient compliance, and better quality of life among patients (10, 11). Unfortunately, research suggests that empathy levels among students decline over time, with increased patient contact (7, 12). For this reason, it is imperative that the pharmacist–patient relationship be enhanced to provide safe and high quality care. In light of this finding, several authors have developed educational exercises that aim to improve empathy levels among pharmacy students (13, 14).

**Figure 1:**
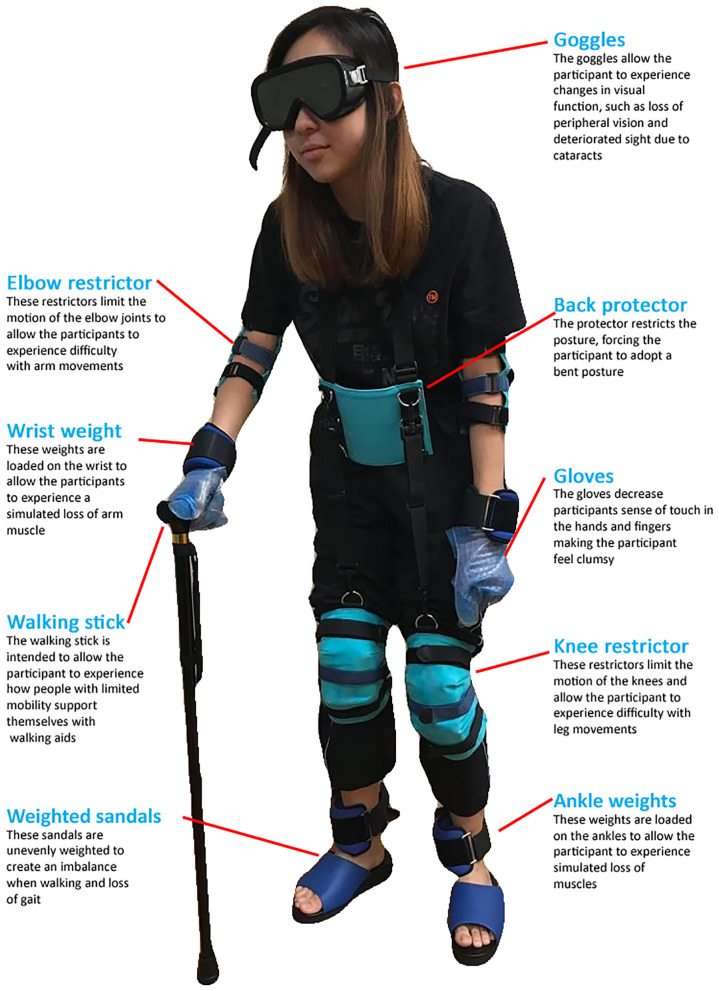
The full setup of aging suit.

Simulation has been suggested as a novel educational approach to teach healthcare students empathy for older adults (15, 16). Simulation suits, such as GERontologic Test suit (17) and Age Gain Now Empathy System (18) were recently introduced, and offer the opportunity for younger people to experience the impairments experienced by older adults. The simulation suit usually consists of a pair of specially designed glasses which simulates opacity of the eye lens and narrowing of visual field; a pair of gloves which simulates decreased sensitivity and grip ability; a weighted vest which increases weight and mimics spinal deformities; elbow and knee wraps which result in restricted mobility and flexibility; and sand bags which are worn on the wrists and ankles to simulate slow movement as well as a pair of unevenly weighted sandals to simulate decreased flexibility and loss of gait (Figure 1).

The use of an immersive aging suit has been examined among nursing students, aimed at enhancing the students' appreciation of the physical and sensory difficulties among older adults. Bennett and colleagues examined how the use of an aging suit impacted health students as to the functional loss and social isolation among older adults (16). In the study, the authors noted that students gained a better understanding of the functional issues and social isolation that are associated with aging after using the aging suit. Lavallière et al. similarly examined the use of an aging suit among younger adults (19) and found that they could relate better to the physical limitations and reduced performance associated with aging.

Pharmacists are trained traditionally to focus on patient counseling especially on medication-related issues, which are commonly seen among older adults who may have to move from independent living circumstances to long-term care (11, 20–22). Nevertheless, this process is often considered as ticking a checklist rather than as a two-way communication that involves the empathy needed for delivery of patient-centered care (23). Cognizant of this limitation, the Bachelor of Pharmacy course at Monash University was redeveloped recently to establish early and regular patient contact and by ensuring that communication and clinical skills courses are co-taught from the very beginning. First-year pharmacy students in the Bachelor of Pharmacy course are taught communication skills in different settings, including communication with older adults, since this is crucial for the students' personal growth, development, and critical reasoning.

To the best of our knowledge, no study has yet evaluated whether using both a simulation exercise and an aging suit will improve pharmacy students' empathy levels, their ability to respond, and to use these skills in patient interactions. This mixed-method open-label randomized controlled study investigated the hypothesis that students' empathy can be enhanced by integrating an aging simulation exercise with a polypharmacy workshop. We hypothesized that students would improve their empathy after using the aging simulation suit compared to those who do not use the suit during their studies.

## Methods

### Study Design and Setting

This study was a randomized, parallel-group, open-label study conducted at Monash University Malaysia between March 2018 and September 2018. The study was registered with ClinicalTrials.gov (NCT04133727).

### Participants and Allocation

All Year 1 Bachelor of Pharmacy students who were enrolled at Monash University Malaysia in 2018 were invited to participate in this study. The program is a 4-years course identical to the course offered at Monash University in Australia. Participants were recruited if they were (1) first-year pharmacy students; (2) enrolled in the Professional Practice I (PHR1011) unit; and (3) were in self-reported good health. Participants who had any current experience of dizziness or numbness in the limbs were excluded from the study.

### Randomization and Masking

Participants were randomized 1:1 to receive either intervention or control using a computer-generated random number table, 2 weeks before the polypharmacy workshop. However, due to the nature of the study, it was not feasible to blind the participants or researchers to intervention allocation upon randomization. The study was approved by the Monash University Human Research Ethics Committee (2017-11714-14826).

### Intervention

Participants randomized to the intervention group were required to wear an aging simulation suit (Nagoya, Japan: Yagami Inc.) and to perform a range of activities that aim to simulate some of the physical disabilities and challenges an older adult may experience in their daily tasks (Table 1). Activities performed include sitting down and getting up from a chair and sofa, reading and filling out a form during a healthcare clinic visit, as well as picking up an object from the floor. These activities were performed in the classroom a week prior to the polypharmacy workshop and took ~10 min to complete per participant. All participants performed the task only once.

**Table 1:** Areas of aging experienced by students and tasks performed.

**Areas examined**	**Task performed**
Visual limitation associated with ocular diseases	The participant was asked to complete a standard demographic questionnaire which was prepared on a clipboard with a pen. After a minute has passed, the participant was requested to pass the form back.
Restricted physical strength including gait, movement and strength	Participants were asked to don the full suit on and walk for 100 m and sit down on a sofa. The participant was then asked to stand up and sit on another stool. Participants were also provided with a comb and asked to comb their hair using their non-dominant hand.
Grip strength and sense of touch	Participants were provided with a piece of paper, which was deliberately dropped and asked to pick it up. This activity was repeated using a 10 cent coin (diameter 1.5 cm)
Balance	Participants were requested to sit down on a high stool and walk up a flight of stairs.

### Polypharmacy Workshop (Control)

As part of the Bachelor of Pharmacy curriculum, all students were required to participate in a polypharmacy workshop with older adults, arranged at week 12 (final week of curriculum) on campus. The session aims to outline the occurrence of polypharmacy among older adults and identify challenges that may arise from managing polypharmacy. The session also provided students with the opportunity to learn effective communication with older adults. For the session, older adults aged 60 years and above from the local community were recruited by the school administrative staff. This age limit was chosen as it is the typical retirement age in Malaysia. These older adults were required to be taking at least five or more medications (average medications per older adult in Malaysia: 6.2 medications) and were willing share their experiences with students during the workshop.

The workshop consisted of a 60-min session with four participants assigned to speak to two older adults. During the session, participants were required to assess and determine the older adults' health literacy; discuss their medicine use; discuss the difficulties they may have been facing in relation to medicine use; and complete a medication list and convey this information to each of the older adults that they had interviewed, with a copy of their list. Academic staff members who were facilitating the session provided a debriefing to students, concentrating on the medication-related issues.

### Data Collection and Evaluation

**Figure 2:**
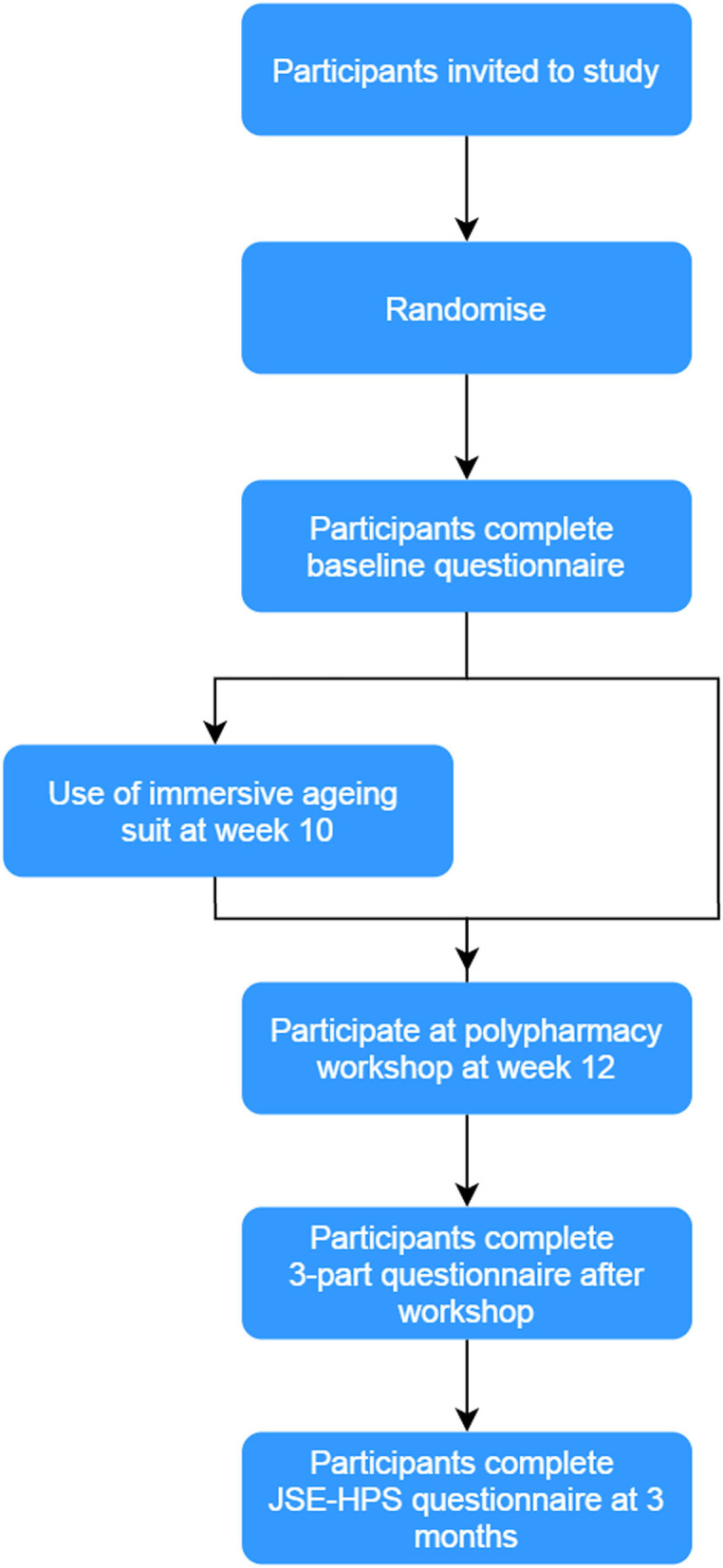
Overview of study. Participants who agreed to the study were randomized 1:1 to either an immersive aging simulation suit and medication review polypharmacy workshop or medication review polypharmacy workshop (control). All participants completed a baseline questionnaire at week 0 and post-workshop questionnaire at weeks 12 and 24.

We collected the participants' demographic characteristics and scores on the Jefferson Scale of Empathy–Health Profession Student (JSE-HPS) (24), at the beginning of their course at week 1 (baseline, Figure 2). JSE-HPS was measured on a seven-point Likert scale which can be summed to generate a total score of between 20 and 140, with a higher score indicative of greater empathy during provider patient encounters. The tool has been validated for use among pharmacy and nursing students to measure empathy (25–27) with good reliability and validity (25).

At the end of the workshop at week 12, all participants completed a three-part questionnaire. The first section assessed the participant's self-perceived impact of the activity toward their attitude, empathy, and understanding of older adults using a 5-point Likert scale with 1 representing none at all to 5 representing a great deal. The second section comprised the JSE-HPS questionnaire, while in the third section, participants were asked to complete an open-ended questionnaire to describe how the task affected their feelings and perceptions toward older adults (refer to Supplementary Materials for list of questions). Participants also completed the JSE-HPS questionnaire again, 3 months after the workshop (week 24).

### Outcomes

The primary outcome of interest was the change in JSE-HPS scores from baseline to the end of the workshop (week 12). Secondary outcomes included the longitudinal impact of the intervention, 3 months after the workshop, and perceived changes in attitude, empathy toward and understanding of older adults.

### Statistical Analyses

Assuming that the intervention would improve participants' empathy scores by 10%, we needed a minimum of 108 participants. This sample size has a 90% power to detect an α of 0.05 between each group, factoring a 20% dropout rate.

Participants' baseline characteristics were summarized using descriptive statistics and presented as mean (standard deviation) for continuous variables and total number (percentages) for categorical variables. The characteristics were compared across groups using the analysis of variance for continuous variables or the χ^2^ test for categorical variables. Following an intention-to-treat protocol, we analyzed the primary outcome with their estimates based upon their randomization allocation using a multivariate general linear model, which controlled for the age and gender, since these variables have been previously suggested as possible confounders (28). The model predicted from the treatment group as well as time interaction, using all available data from baseline and follow-up time points. To consider the potential impact of missing data, we imputed missing outcome data, assuming that data were missing at random. We performed sensitivity analyses for the primary outcome by repeating the analysis including only participants who had completed the survey. All analyses were performed using the IBM SPSS Statistics, version 25.0 (Armonk, NY: IBM Corp).

All open-ended responses were coded into themes following the principles of Miles et al. (29). Using this method, the texts were coded into nodes, based upon questions posed to participants. The codes were generated independently by two authors (SWHL and PLT). Coding was done on paper using a coding matrix developed by the researchers on Microsoft Word. A discussion was held between both researchers and conclusions were drawn by identifying for category clusters that led to the development of overarching themes and sub-themes. In cases of disagreement, both authors discussed the matter until a consensus was achieved.

## Results

**Figure 3:**
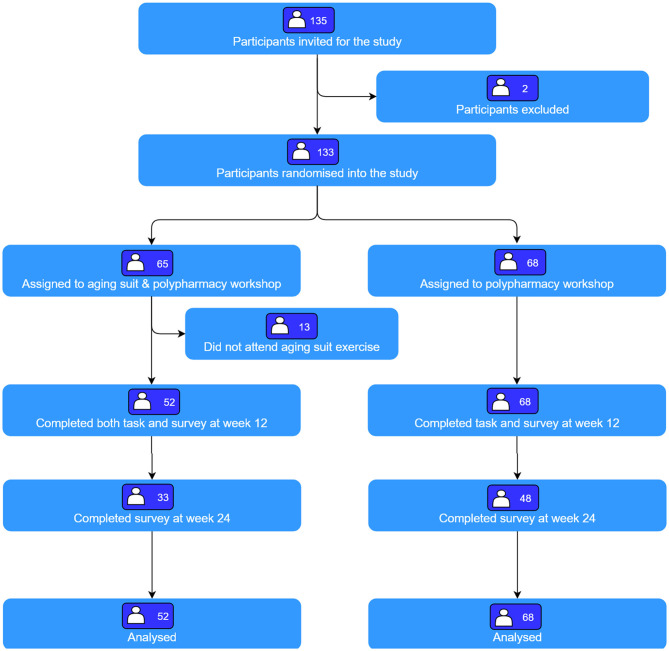
Participant flow through the study.

Of the 135 participants approached, 133 participants were randomized into the study (Figure 3). A total of 65 participants were randomized to intervention and 68 to control. Of these, 13 participants randomized to intervention were excluded as they did not take part in the aging suit activity. Thus, the analyses included 68 participants randomized to control and 52 to intervention. Participants had a mean age of 19.5 (0.7) years and were mostly female (77.5%, *n* = 93). The demographics of both groups were similar at baseline (Table 2).

**Table 2:** Demographic characteristics of participants in the study.

**Characteristics**	**Aging suit with workshop (*n* = 52)**	**Workshop only (*n* = 68)**	**Overall (*n* = 120)**	***p*-value**
Mean (SD) age (years)	19.5 (0.7)	19.5 (0.8)	19.5 (0.8)	0.81
Gender, *n* (%)^*^				0.57
Male	13 (25.0)	14 (20.6)	27 (22.5)	
Female	39 (75.0)	54 (79.4)	93 (77.5)	
Pre-university entry qualification, *n* (%)^*^				0.21
Foundation studies	31 (59.6)	34 (50.0)	65 (54.2)	
GCSE A-level or equivalent	19 (36.5)	23 (33.8)	42 (35.0)	
Matriculation or equivalent	1 (1.9)	8 (11.8)	9 (7.5)	
Unified examination certificate	1 (1.9)	3 (4.4)	4 (3.3)	
Mean (SD) JSE-HPS	111.5 (13.6)	111.9 (11.3)	111.8 (12.2)	0.86

* *Chi-square test was used to determine significance, defined as p < 0.05*.

*JSE-HPS, Jefferson Scale of Empathy-Health Profession Student Version*.

### Primary Outcome

In the pre-specified intention-to-treat analysis, both groups reported improvements in JSE-HPS scores from baseline, with a mean improvement of 1.7 (14.5) points in the intervention group compared to 1.2 (9.4) points in the control group, but this did not reach statistical significance. No significant differences between participants' JSE-HPS scores were noted between those randomized to intervention and control (mean difference: −0.5 points; 95% confidence interval: −3.83 to 4.83; *p* = 0.81).

### Secondary Outcomes

**Figure 4:**
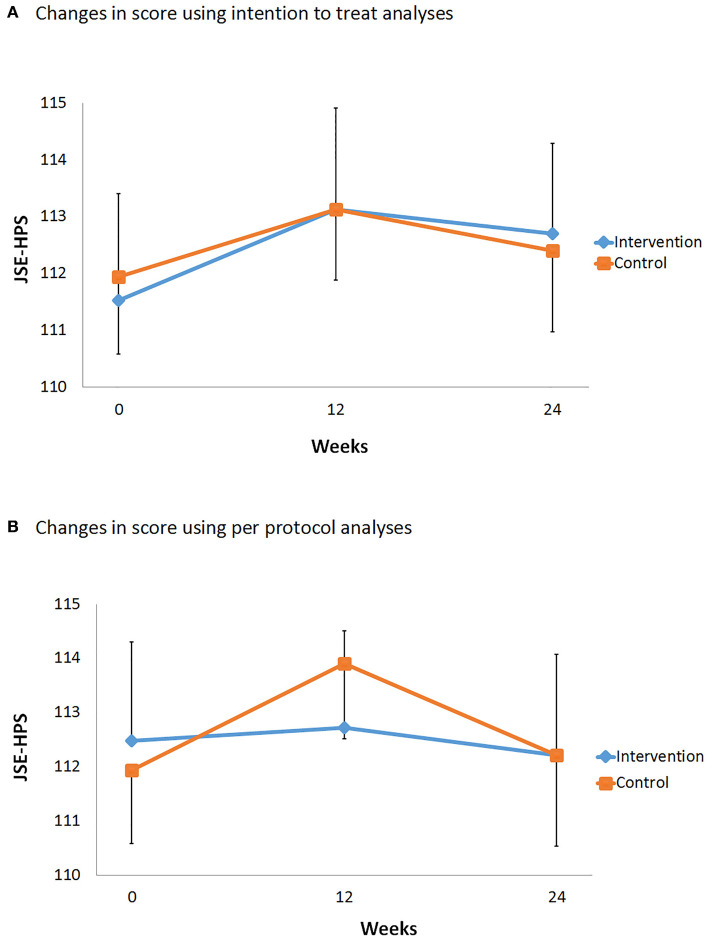
Comparison of Jefferson Scale of Empathy–Health Profession Student score over time. The intervals represent standard error for each group. **(A)** Intention-to-treat analysis. **(B)** Per-protocol analysis.

**Figure 5:**
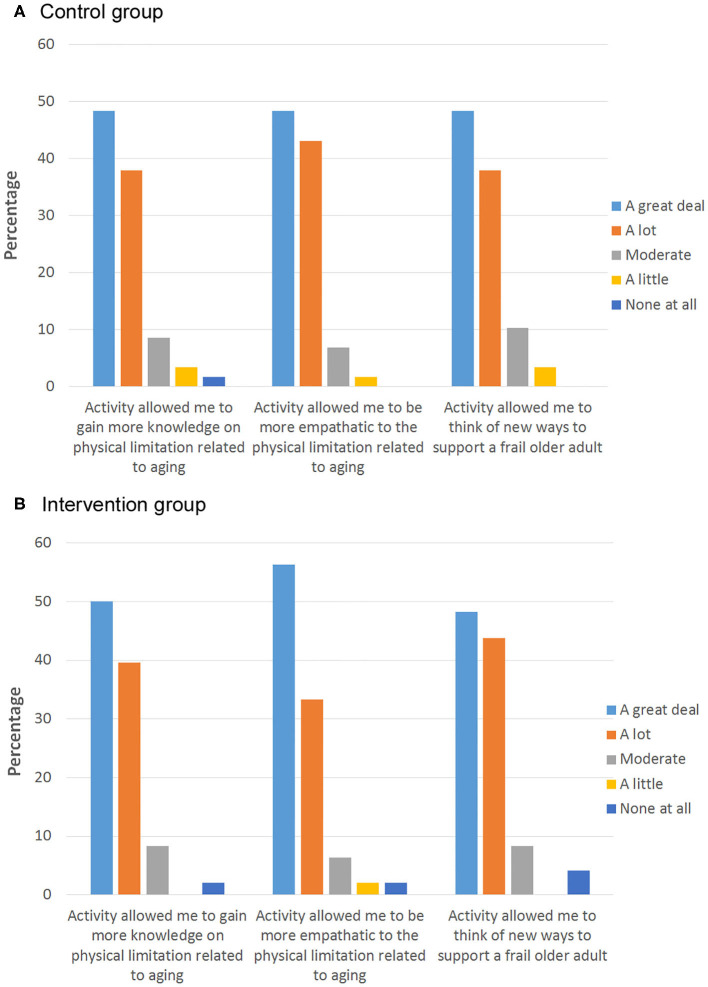
Participants' reported change in attitude, empathy level, and understanding of older adult in the **(A)** control group and **(B)** intervention group after the activity. Bar chart represents the percentage of participants.

Three months after the workshop activity, the JSE-HPS scores fell back to baseline levels in the intervention and control groups. No significant difference were noted in JSE-HPS scores between both groups (mean difference:-0.3; 95% CI: −5.16 to 5.76, *p* = 0.30; Figure 4A). The analysis of participants who completed the study (per-protocol analysis) showed no significant differences between both groups after the intervention at week 12 and 24 (Figure 4B). At the end of the workshop, participants' self-rated knowledge and understanding on the physical limitations of aging were similar between both groups (*p* = 0.79). No difference in self-reported attitudes about the importance of empathy (*p* = 0.70) as well as ability to support older adults (*p* = 0.34) between groups were also noted (Figure 5).

### Qualitative Data

Open-ended responses from 105 participants (87.5%) identified several common themes that spanned across both groups. These were: (1) “Lending an ear”; (2) A sense of respect; and (3) Understanding the emotions. We also identified an additional theme in the intervention group; namely a more realistic view of aging (Table 3).

**Table 3:** Key themes identified in the current study.

**Main themes**	**Exemplar quotes**
Lending an ear	•“*Patients' trust can be established if we are willing to empathize with them. Hence, we need to accept their feelings and frustrations, and come up with a solution within [the] permitted time”* Student 10, Control •“*…[I feel the need to] learn more languages to avoid communication barrier”* Student 61, Intervention
A sense of respect	•“*… but rather a sense of inferiority and humility, knowing that the experience and skills that I have now are incomparable to what [of] theirs, it motivates me to learn humbly from those who are ahead in age and experience of me. Also I would like to behave like them when I am approaching their age, still as motivated and optimistic in spite of their past experience”* Student 10, Control •“*… and respect them as someone who has been through life more than I did and someone that I [can] look up to and have a great deal of respect”* Student 98, Intervention
Understanding the emotions	•“*I used to lack the patience when dealing with older adults but are more willing and more patient to listen and talk to them”* Student 61, Control •“*I feel sentiment not only because the physical illness of the elderlies but also their emotions. One of the ladies is a divorcee and she had to take care of herself alone. The other lady had endometriosis which makes her infertile. Their stories make me feel sad because of the hard times and suffering they've gone through”* Student 115, Control
Realistic view of aging	•“[*I*] *felt a lot of limitation [which] I had never felt before. These limitations are something you are cannot control. You feel weak and helpless because you cannot change or alter [these limitations]*” Student 59, Intervention •“*My ability to see and feel was significantly worsened [by wearing the suit] and it felt very difficult to do things. The thing that surprised me most is that every old person is experiencing this and there are still people that don't understand how difficult it is for elderly people to do things”* Student 66, Intervention •“*I had blurred vision and had less sensitivity in my palms. I was most surprised I could not really feel much using my palms and it was hard to do simple things like picking up a coin”* Student 67, Intervention

### “Lending an Ear”

Participants' from both groups described the importance of taking time to listen to an older adult. Participants described how the polypharmacy workshop had provided them with an opportunity for a practical interaction with older adults and that it had revealed the importance of active listening. They described how each older adult they spoke with had their own story to tell and how a rushed and hurried patient-provider interaction was detrimental to their understanding of what would be needed to provide better patient care.

Participants also described the importance of having good communication skills and the importance of using open-ended questions during an interaction with older adults. Some participants also reflected upon the importance of learning other languages and dialects to improve their interactions with older adults.

### A Sense of Respect

The activities also evoked a sense of respect among participants toward older adults. Participants from both groups described that the activities made them realize that older adults face many challenges in their lives, especially in relation to medication usage. Participants also mentioned how these older adults had shared their life experiences and noted how they should always remember to take into consideration patients' feelings during any interactions.

### Understanding the Emotions

Participants mentioned that the activity provided them with a practical interaction and helped them understand the importance of empathy in healthcare. The activity gave them a unique opportunity to speak to older adults and understand the emotions that these older adults experience. Through the activity, participants mentioned that they could relate to using different communication techniques, including verbal and non-verbal communication as being essential skills for healthcare providers. Participants also reflected on how the activity would affect their future communication with older adults, such as speaking more slowly, using a suitable volume, and using appropriate tones.

### Realistic View of Aging

Participants in the intervention group described how the simulation suit exercise provided them with a deeper insight into both physical and emotional issues related to older adults. Nearly all participants described how the suit made them realize why older adults had mobility issues, especially related to walking. Other changes reported included physical limitations resulting in difficulty in getting up from a sofa, walking more slowly, as well an inability to read product information that uses small print. Some participants also reflected upon this and how it related to their interactions with their own grandparents.

Participants also described the loss of sensory feedback they felt when they wore the suit and could relate the difficulty they experienced especially when holding a pen and trying to fill in a form. Participants lamented about the difficulty with vision caused by changes to their eyesight during their experience and had great admiration and respect for older adults who could drive and read despite these limitations. Some participants mentioned that in the future, they would make changes to their practice as a pharmacist including writing in larger fonts, speaking clearly, and smiling at older adults.

## Discussion

The concept of using simulation suits is relatively new and is increasingly being examined as a learning tool for healthcare students (15, 16, 19), but use of simulation suits had not been studied in a randomized controlled cohort of pharmacy students. Our study found that the immersive aging simulation suit did not significantly improve participants' self-rated empathy levels compared to a polypharmacy workshop only. This may be due to the self-reported measure using JSE-HPS which we had utilized. Riess et al. have previously examined how empathy training could be improved in a cohort of resident physicians assigned to an augmented empathy training protocol, compared with control (30). They found no difference in the self-reported JSE scores in their cohort, but patient-rated empathy scores were significantly higher in the intervention group, suggesting that patient and self-assessment may not measure the same phenomenon (31). In our study, we noted similarly that results from our qualitative results are in contrast to those from the JSE-HPS scores. However, we believe that this is only one part of a larger puzzle as the open-ended responses of participants in the intervention showed improvements in a more important component that is rarely examined: personal growth.

Students expressed that it is sometimes difficult to relate the decreased physical function and sensory feedback experienced by an older adult (32). While the experimental tasks in this study were not a perfect replica of actual aging, the study showed that participants who wore the suit experienced changes in task performance, consistent with those associated with aging. The impact of the suit was most pronounced for the tasks related to flexibility, which was not experienced by the control group who had only interacted with older adults. In addition, we observed an increased awareness among participants in the intervention of some of the changes that are associated with aging, such as the changes in vision.

### Implications for Future Research

The use of simulation as an educational methodology for teaching empathy is being examined increasingly by researchers (33). Our study suggests that the use of the immersive simulation suit can be a valuable adjunctive learning modality to enhance the understanding of pharmacy students of the physical limitations experienced by older adults. The use of the suit may have resulted in some students experiencing role reversal which students felt as though they were really older and, thus, could be an important mechanism in enhancing the empathy levels among students and should thus be explored further in the future. We believe that this activity also provides students with a personal and professional learning opportunity to think about their attitude toward older adults. The richness from our mixed-method study helped to clarify a range of factors that can improve or impede students' empathy levels. Students expressed that they had a more positive experience with the use of the simulation suit as suggested in our open-ended questionnaire responses received.

This technique can easily be replicable and adopted by the other medical professions, such as doctors, dentists, and nurses to improve the understanding of the potential physical limitations of an older adult. This practice can be advantageous for all healthcare professionals, since they are more likely to receive positive feedback from patients, which can be rewarding. Moreover, improved communication and trust in clinical settings can also have tangible effects, such as better patient compliance with treatment regimens and higher patient satisfaction (34).

### Limitations

Our study has some important limitations which need to be acknowledged. Firstly, the age simulation suit does not attempt to simulate any cognitive changes associated with aging, such as dementia. In addition, we did not control for other confounders which may affect the overall experience of students, such as the level of fitness of each student. The suit was only worn for a very short period of time (~10 min), and each of the tasks was only performed once, which may not allow for each participant to experience fully the physical decline that an older adult experiences in his/her daily life. As such, future studies should ideally examine the impact of using an aging suit over an extended period of time, similar to how medical students would wear a cast for weeks to simulate a broken arm (35). This could be supplemented with a self-reflective writing exercise, which helps the observer to become more aware of his/her own emotion and subsequently improve his/her ability to be more empathetic toward another individual (36).

Some of the student participants could be living with older adults, which was not taken into consideration for the analysis. Future iterations of this activity would also take into account the students' lived experience with older adults. Our study may also be underpowered to detect the changes we had anticipated because of the small sample size that we had recruited. The study was also conducted among first-year pharmacy students and thus cannot be generalized to students from other year levels. Finally, our study assessed only empathy using a first person rating using the JSE-HPS scale, which may have resulted in the lack of significant findings reported in this study.

In summary, with an increasing population of older adults, we feel that it is of great benefit that healthcare students can appreciate what it feels like to be an older adult. The immersive aging suit can be a useful adjunctive measure for teaching students about the physical challenges faced by older adults. However, a larger study on various healthcare students' personalized patient care and overall health outcomes should be conducted in the future.

## Data Availability Statement

All datasets generated for this study are included in the article/Supplementary Material.

## Ethics Statement

The studies involving human participants were reviewed and approved by Monash University Human Research Ethics Committee (2017-11714-14826). The patients/participants provided their written informed consent to participate in this study. Written informed consent was obtained from the individual for the publication of any potentially identifiable images or data included in this article.

## Author Contributions

SL designed the study and conducted data analysis. SL and P-LT assisted in the data analysis and drafting of the manuscript. All authors approved the final version of the manuscript.

## Conflict of Interest

The authors declare that the research was conducted in the absence of any commercial or financial relationships that could be construed as a potential conflict of interest.
