# Supplementary material for: “Suiting Up” to Enhance Empathy Toward Aging: A Randomized Controlled Study
Source: Front Public Health. 2020 Aug 25;8:376. doi: 10.3389/fpubh.2020.00376 (PMC7477349; doi:10.3389/fpubh.2020.00376)
Supplement: Supplementary file 1 [file Table_1.DOCX]

## List of semi-structured open ended questions

1. What did you learn from participating in the activity?
2. Which part of the activity that you felt most effective?
3. Describe some of the feelings or emotion after you participated in the activity
4. How has your attitude changed towards older adults? How did it change?
5. Now that you have participated in the activity, how do you think it will affect your practice as future pharmacists
6. What are some of the changes you plan to make in your practice?
