# Supplementary material for: “Suiting Up” to Enhance Empathy Toward Aging: A Randomized Controlled Study
Source: Front Public Health. 2020 Aug 25;8:376. doi: 10.3389/fpubh.2020.00376 (PMC7477349; doi:10.3389/fpubh.2020.00376)
Supplement: Supplementary file 2 [file Data_Sheet_1.PDF]

Checklist: Simulation-based Research Extensions for the CONSORT Statement

Checklist completion and submission required for all randomized controlled trials

**Item**	**Item no**	**CONSORT Description (Randomized, controlled trials)**	**Extension for Simulation-based Research**	**Reported on Page #**
**Title and abstract**	1a,1b	1a: Identification as a randomized trial in the title<br>1b: Structured summary of trial design, methods, results, and conclusions	In abstract or key terms the MESH or searchable keyword term must have the word "simulation" or "simulated".	☐ 1<br>1-3
**Introduction**				
Background	2a, 2b	2a: Scientific background and explanation of rationale<br>2b: Specific objectives or hypotheses	Clarify whether simulation is *subject of research* or *investigational method for* research.	☐<br>4-6
**Methods**				
Trial Design	3a, 3b	3a: Description of trial design (such as parallel, factorial) including allocation ratio<br>3b: Important changes to methods after trial commencement (such as eligibility criteria), with reasons		☐<br>7
Participants	4a, 4b	4a: Eligibility criteria for participants<br>4b: Settings and locations where the data were collected		☐<br>7
Interventions	5	The interventions for each group with sufficient details to allow for replication, including how and when they were actually administered	Describe the theoretical and/or conceptual rationale for the design of each intervention.<br>Clearly describe all simulation-specific exposures, potential confounders, and effect modifiers.	☐<br>7-8
Outcomes	6a, 6b	6a: Completely defined pre-specified primary and secondary outcome measures, including how and when they were assessed<br>6b: Any changes to trial outcomes after the trial commenced, with reasons	In describing the details of methods of assessment, include (when applicable) the setting, instrument, simulator type, timing in relation to the intervention, along with any methods used to enhance the quality of measurements.<br>Provide evidence to support the validity and reliability of assessment tools in this context (if available).	☐<br>9
Sample size / Study size	7a, 7b	7a: How sample size was determined<br>7b: When applicable, explanation of any interim analyses and stopping guidelines		☐<br>9
Randomization: Sequence generation	8a, 8b	8a: Method used to generate the random allocation sequence<br>8b: Type of randomization; details of any restriction (such as blocking and block size)		☐<br>7
Randomization: Allocation concealment mechanism	9	Mechanism used to implement the random allocation sequence (such as sequentially numbered containers), describing any steps taken to conceal the sequence until interventions were assigned		☐<br>7
Randomization: Implementation	10	Who generated the random allocation sequence, who enrolled participants, and who assigned participants to		☐<br>7

		interventions		
Blinding (masking)	11a, 11b	11a: If done, who was blinded after assignments to interventions (for example, participants, care providers, those assessing outcomes) and how<br>11b: If relevant, description of the similarity of interventions	Describe strategies to decrease risk of bias, when blinding is not possible.	☐ 7
Statistical Methods	12a, 12b	12a: Statistical methods used to compare groups for primary and secondary outcomes<br>12b: Methods for additional analyses, such as subgroup analyses and adjusted analyses	Clearly indicate the unit of analysis (e.g. individual, team, system) and identify repeated measures on subjects, and describe how these issues were addressed.	☐ 9-10
**Results**				
Participant flow (a diagram is strongly recommended)	13a, 13b	13a: For each group, the numbers of participants who were randomly assigned, received intended treatment, and were analysed for the primary outcome<br>13b: For each group, losses and exclusions after randomization, together with reasons		☐ 11 & Figure 1
Recruitment	14a, 14b	14a: Dates defining the periods of recruitment and follow-up<br>14b: Why the trial ended or was stopped		☐ 11
Baseline data	15	A table showing baseline demographic and clinical characteristics of each group	In describing characteristics of study participants, include their prior experience with simulation and other relevant features as related to the intervention(s).	☐ 11
Numbers analyzed	16	For each group, number of participants (denominator) included in each analysis and whether analysis was by original assigned groups		☐ 11
Outcomes and estimation	17a, 17b	17a: For each primary and secondary outcome, results for each group, and the estimated effect size and its precision (such as 95% confidence interval)<br>17b: For binary outcomes, presentation of both absolute and relative effect sizes is recommended	For assessments involving more than one rater, inter-rater reliability should be reported.	☐ 12
Ancillary analyses	18	Results of any other analyses performed, including subgroup analyses and adjusted analyses, distinguishing pre-specified from exploratory		☐ 12-16
Adverse Events	19	All important harms or unintended effects in each group (for specific guidance see CONSORT for harms)		☐ NA
**Discussion**				
Limitations	20	Trial limitations, addressing sources of potential bias, imprecision, and, if relevant, multiplicity of analyses	Specifically discuss the limitations of simulation-based research.	☐ 18
Generalizability	21	Generalizability (external validity) of the trial findings	Describe generalizability of simulation-based outcomes to patient-based outcomes (if applicable).	☐ 17-18
Interpretation	22	Interpretation consistent with results, balancing benefits and harms, and considering other relevant evidence		☐ 18
**Other Information**				
Registration	23	Registration number and name of trial registry		☐ NA
Protocol	24	Where the full trial protocol can be accessed, if available		☐ NA
Funding	25	Sources of funding and other support (such as supply of drugs), role of funders	List simulator brand and if conflict of interest for intellectual property exists.	☐ 19
